# Riedel’s Thyroiditis with Intense FDG Uptake Demonstrated on FDG PET/CT

**DOI:** 10.4274/mirt.98598

**Published:** 2015-02-15

**Authors:** Robert Mansberg, Rosamma Bency, Lily Shen, Chuong Bui, Kris Park

**Affiliations:** 1 Nepean Hospital, Clinic of PET and Nuclear Medicine, Penrith, Australia; 2 Discipline Medicine University, Sydney, Australia

**Keywords:** Riedel’s thyroiditis, FDG PET/CT, Retroperitoneal Fibrosis

## Abstract

A 39 year old female presented with rapidly enlarging goitre, minimal obstructive symptoms and no constitutional symptoms. Clinical examination revealed diffusely enlarged, firm, non-tender thyroid gland. Biochemical investigations showed subclinical hypothyroidism, positive thyroid antibodies and unremarkable inflammatory markers. Ultrasound examination and CT scan of the neck were suspicious of Riedels thyroiditis. The patient was referred for a FDG PET scan to evaluate for systemic fibro-inflammatory process or lymphoma. Subsequent core biopsy of the thyroid gland demonstrated a chronic inflammatory process with fibrosis consistent with Riedels thyroiditis. A FDG PET/CT study showed diffuse FDG uptake in the thyroid gland and no abnormal retroperitoneal FDG uptake elsewhere to suggest active retroperitoneal fibrosis. The goitre reduced in size with thyroid hormone replacement and steroids, however the patient was lost to follow up.

## Abstract (tr)

Otuz dokuz yaşında bir kadın hasta, hızlı büyüyen bir guatr tablosu, minimal obstrüktif belirtiler ile başvurdu. Ek yakınmaları yoktu. Fizik muayenede tiroid bezi diffüz olarak büyümüş, sert ve ağrılı idi. Biokimyasal tetkiklerde subklinik hipotiroidi ve positif tiroid antikorları saptanırken inflamasyon belirteçleri yüksek değildi. Ultrason ve boyun BT görüntüleme tetkikleri Riedel tiroiditini düşündürmekteydi. Hasta, sistemik bir fibro-inflamatuvar hastalık ya da lenfoma yönünden değerlendirilmek üzere FDG PET görüntülemeye yönlendirildi. Bunu takiben yapılan tiroid bezi biyopsisi Riedel tiroiditi ile uyumlu olan, fibrozisin eşlik ettiği kronik inflamatuvar bir süreci gösterdi. FDG PET/BT incelemesi tirod bezinde diffüz artmış FDG tutulumunu gösterirken, aktif retroperitoneal fibrozu düşündürecek anormal bir retroperitoneal FDG tutulumu mevcut değildi. Tiroid hormon replasmanı ve steroidlerle guatr boyutu küçültüldü, ancak hastanın ileri takipleri yapılamadı.

## INTRODUCTION

Riedel’s thyroiditis is a rare entity of unknown aetiology, recently included in the spectrum of IgG4-related disease (1) in which fibrous tissue replaces the thyroid gland and tends to extend into the surrounding tissues (2). An association exists between Riedel’s thyroiditis and multifocal idiopathic fibro-sclerosis, which includes retroperitoneal fibrosis, pseudo-tumour of the orbit, sclerosing cholangitis, and mediastinal fibrosis (2). A case of Riedel’s thyroiditis demonstrated on FDG PET is described.

## CASE REPORT

A 39-year-old female presented with signs, symptoms and investigations suggestive of infiltration of the thyroid. In order to further evaluate this process and to assist differentiating lymphoma from Riedel’s Thyroiditis, the patient was referred for a FDG PET/CT. The study demonstrated intense uptake throughout the thyroid gland, which was not specific and subsequent biopsy confirmed a fibrotic disorder consistent with Reidel’s thyroiditis. The patient had a reduction in size of the goitre to thyroid hormone replacement and corticosteroids but was lost to followup.

**Figure 1:**
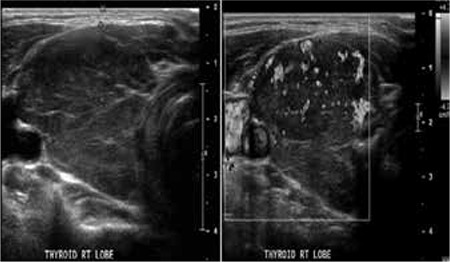
Thyroid ultrasound demonstrated diffusely hypo echoic, asymmetrically enlarged thyroid gland with diffusely increased vascularity, but with no discrete focal mass lesions (only right thyroid lobe shown).

**Figure 2:**
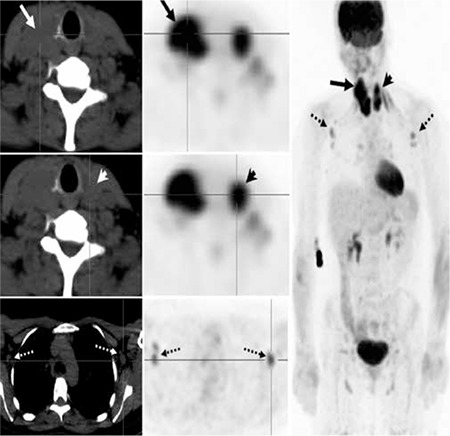
Axial image from neck CT demonstrated asymmetrically enlarged thyroid gland (right left) with lobulated appearance and mild compression of the right side of the trachea (Solid white arrow).

Thyroid ultrasound demonstrated diffusely hypo echoic, asymmetrically enlarged thyroid gland (right>left) with diffusely increased vascularity, but with no discrete focal mass lesions. Figure 1 (only right thyroid lobe shown): Contrast enhanced diagnostic CT of the Neck, chest, abdomen and pelvis was performed. Axial image from neck CT demonstrated asymmetrically enlarged thyroid gland (right>left) with lobulated appearance and mild compression of the right side of the trachea (Solid white arrow) (Figure 2). There was no evidence of direct involvement of surrounding structures in the neck or of retroperitoneal fibrosis.

Axial and MIP images from whole-body FDG-PET/CT (64 slice Phillips TF Gemini, 1 hour post injection of 6.6 mCi (245 MBq) of F-18 FDG.) demonstrated diffuse intense heterogeneous FDG uptake (SUV max 15.4, solid arrows) in an asymmetrically enlarged thyroid gland (right>left) (Figure 2). Mild to moderate FDG uptake (SUV max 2.2) was noted in non enlarged lymph nodes in a number of nodal stations on both sides of the diaphragm, most prominent in the axillae (dotted arrows), and also in bilateral neck, mediastinum, bilateral pulmonary hila, bilateral external iliac and bilateral inguinofemoral regions The nodal FDG uptake was considered nonspecific and could represent inflammatory or reactive aetiology. A differential diagnosis of lymphoma was also considered in addition to the clinical diagnosis of Riedels thyroiditis. Subsequent core biopsy of the thyroid gland illustrated chronic inflammatory process with fibrosis consistent with Riedels thyroiditis. There was no evidence of increased FDG uptake in the retro peritoneum or elsewhere to suggest a systemic fibro-inflammatory process. Progress imaging post treatment with steroid treatment was not performed as the patient was lost to follow up.

**Literature Review and Discussion**

Pathologically, Riedel’s thyroiditis is characterised by replacement of normal thyroid tissue by fibrotic connective tissue, with obliteration of normal thyroid architecture and involvement of adjacent tissues by direct extension through the thyroid capsule. Diffuse or partial involvement of the gland may occur. There is absence of skin and lymph node involvement. Clinically, the thyroid gland is enlarged and hard but non tender and frequently involves a previously existing goitre. Direct extension causes compression of the trachea and the oesophagus, resulting in dyspnoea and dysphagia. The nodal FDG uptake in this patient is not a typical feature of IgG4 disease (1) and may represent reactive/inflammatory change or possibly other pathology (these nodes were not biopsied).

The natural history of Riedel’s thyroiditis is most likely self-limiting; however, treatment can include steroids, as well as surgical excision of all or part of the gland to alleviate compressive symptoms (1). The differential diagnosis includes carcinoma, lymphoma, and Hashimoto’s thyroiditis, but the extent of the fibrosis in these disorders is much less.

Computerised tomography is used to assess the extent of fibrosis, in which the thyroid appears hypodense, and invasion of nearby tissues is occasionally present, with slight enhancement after contrast medium administration. On magnetic resonance imaging, the thyroid is reported to be hypointense on T1 and T2 weighted images. Ultrasound features, such as vascular encasement seem to be specific to this rare disease (3,4,5). Diffuse intense Gallium-67 uptake has been previously reported in Riedel’s thyroiditis (3).

Diffusely increased FDG uptake in the thyroid with FDG PET study is not specific for Riedel’s thyroiditis and may be found incidentally (6,7). It has been described as an indicator of chronic thyroiditis. Bilateral thyroidal uptake of FDG can be found in normal variants and subjects with various thyroid disorders, showing varieties of uptake patterns. Diffuse intense uptake and higher SUV levels are a clue to a diagnosis of chronic thyroiditis, especially for those with hypothyroidism (8,9). FDG PET can be useful in establishing the diagnosis of associated multifocal fibrosclerosis, evaluating the activity of the inflammatory process and may be of value in evaluating the effects of steroid therapy (6,7).
